# Taboos among general population about dental extraction in Sangli region: A prospective study

**DOI:** 10.6026/973206300211714

**Published:** 2025-05-31

**Authors:** Sanjay Byakodi, Mushtak Khan, Jyoti Biradar, Kishan Dudhat, Nidhhi Bihani, Swati Priya

**Affiliations:** 1 Department of Oral and Maxillofacial Surgery, Bharati Vidyapeeth (Deemed to be University) Dental College and Hospital, Sangli, Maharashtra, India; 2 Intern, Bharati Vidyapeeth (Deemed to be University) Dental College and Hospital, Sangli, Maharashtra, India

**Keywords:** Taboos, extraction, zandu balm, vicks

## Abstract

Indians reside in rural areas. Ancient Indian taboos and ideologies have a negative correlation with preventative dental health behaviours; the goal of this study was to identify the current frequency of these beliefs and cultural taboos around dentistry. The taboos and myths related to dental extraction were considered. 33% believe that eye sight will be affected while 53% believed that extracted teeth should be buried. 66% considered that extraction should be done only when tooth is shaking and around 59% considered placement of medicaments like zandu balm or vicks to relieve dental pain.

## Background:

Healthy teeth are not nearly as important as oral health. One cannot separate social environment from health. An important factor in a person's social, economic and personal growth is their dental health [1]. Health is significantly impacted by social and economic issues just as much as by medical interventions. Historically, healing and sickness were addressed via spiritual and religious practices rather than modern medicine. This is a result of the ancient civilization's ignorance [2]. A taboo refers to a strong social prohibition, particularly in urban settings, associated with any aspect of human activity or social tradition deemed sacrosanct and prohibited by moral and religious beliefs [3]. Cultural taboos or beliefs have shaped most people's lives for centuries [4]. The welfare of society is harmed by these bad habits and behaviors [5]. A group of people's shared stories that are an integral element of their cultural identity are called myths. They have a significant impact on people's lives and ways of living, which includes how they seek medical attention when ill. In order to provide patients and healthy individuals with great care and health education, it is vital to understand the myths and misconceptions surrounding oral disorders [6, 7]. Most Indians reside in rural areas, it has been discovered that ancient Indian taboos and ideologies have a negative correlation with preventative dental health behaviors [8]. A myth is a widely held mistake, erroneous belief, or fictionalized interpretation of a person or object that has no bearing on reality. There are countless myths connected to various objects and people all throughout the world. Human ignorance and imagination about things they do not know give rise to myths. A person may believe a myth for a variety of reasons, such as ignorance or the cultural, quasireligious, educational and structural aspects of their society [9].

Oral disorders are quite common worldwide, impacting a substantial segment of the global populace. Oral illnesses disproportionately affect the underprivileged and impoverished population. Most members of this underprivileged group lack literacy. Their understanding of the significance of oral health is usually low [10]. India is a developing nation that has numerous obstacles in providing for the health requirements of its citizens. The vast majority of Indians live in rural regions. People from a variety of cultural and religious backgrounds make up the Indian population. People attend hakim (local traditional practitioners) rather than doctors because they believe in spiritual healing and complementary therapies. Each of these elements has an impact on the myths that are most common in Indian society about health. Health and illness are influenced by culture in unique ways. Dentistry is not an exception to the rule that all medical specialties are influenced by culture. A population's health results are significantly influenced by its society and culture, which are connected to behavioural patterns [11]. These taboos and beliefs are gradually vanishing with the advancement of education, yet they still exist and are frequently seen. These cultural precepts also apply to the dental profession. There have been many different superstitions around teeth and tooth aches; the more well-known ones are covered in this study. There is a negative relationship between the population's preventative dental health practices and traditional Indian beliefs and taboos [12]. Therefore, it is of interest to identify the frequency of these beliefs and cultural taboos on dentistry among Indians in rural area.

## Methods and Materials:

A questionnaire study was conducted among General population in Sangli city to assess taboos among general population about dental extraction in Sangli region. The purpose of this assessment was to identify the current frequency of beliefs and cultural taboos around dentistry that are prevalent among Indian to assist and shape future health programs and raise awareness of dental issues. The study duration was about three months. The participants were selected based on the following inclusion criteria:

[1] Patients aged between 20 to 60 years.

[2] Participants who are willing to participate in the study.

[3] Patient affected with psychological issues and patients not willing for consent are excluded from the study.

The input parameter for sample size calculation used as follows: 90% power of the study, alpha error 0.05, effect size 0.3 (small) and degree of freedom as 5. The calculated sample size was 100 using G* Power software version 3.1.9.2 (Heinrich Heine University, Düsseldorf). The final considered sample size for the study was around 110. The convenient sampling technique was used in the study. A structured, self-administered, close-ended questionnaire was designed to collect the data which consisted of four parts and comprised of 21 questions related to assess taboos among general population about dental extraction in Sangli region. The first part consisted of demographic data such as age, gender, education, occupation and the second, third and fourth part consisted of questions based on taboos among general population about dental extraction in Sangli region respectively. The questionnaire was prepared using Google forms (Google LLC, Mountain View, California, United States) and the link was distributed to the selected participants via e-mail, WhatsApp number and other social media platforms (Instagram, Telegram, etc.). The reliability statistics were calculated and Cronbach alpha value was 0.685. A brief introduction about the study was given and informed consent was also taken from all the participants. Data collected were entered in a spreadsheet (Microsoft Excel, 2016). Statistical analysis was done using descriptive statistics (number and percentage). SPSS (Statistical Package for the Social Science) 23.0 version software (IBM Chicago, Illinois, United States). The p-value was set at 0.05 or 5%.

## Results:

In the Table 1, there were a total of 100 participants between 18 to above 45 years of age. About 45 participants were male and 55 were female. Out of 100 participants, 36 participants were educated and 64 participants were uneducated. Among them 26 were employed and rest 74 participants were unemployed. In Table 2, around 33% of participants believe that eye sight will be affected due to extraction of teeth while 29 % of participants disagree and rest 38% of participants had no opinion regarding the same. Around 53% of participants believed that extracted teeth should be buried while 47% of them disagreed. In Table 3, 34% of participants think extraction of root stumps is not necessary. Around 58% of participant believed that head bath after extraction increases pain. About 51% of participants believe that extraction is the only treatment for tooth pain. About 66% participants considered that extraction should be done only when tooth is shaking and around 59% of total study population considered placement of medicaments like zandu balm or vicks to relieve dental pain.

## Discussion:

Every individual has a different cultural background and socioeconomic status which creates a significant impact on the beliefs and oral health of the population. As India is a vast country, we tried to focus on taboos prevalent in our region impacting the oral health and overall wellbeing of the individuals. So, we conducted a prospective study on taboos among general population regarding dental extractions in Sangli district of Maharashtra. A set of 15 questionnaires were given to them and this study's purpose was to evaluate the percentage of people who still believe the taboos regarding dental extraction and their willingness towards dental treatment as well as tooth extraction [13]. In the present study, We Included individuals of different age groups in which 28% of individuals were between 18-30 years of age, 43% were between 31-45 years of age and 29% of individuals were more than 45 years of age, so as to get a broad idea about their thinking and myths prevalent among them. The middle age group was more interested in this survey indicating increasing concern for oral health among these populations and prevalence of dental issues among them. There was almost an equal distribution between Males and Females. We took this survey to gain an idea among uneducated and unemployed individuals along with educated and employed ones. In this study majority of the population were not very sure whether eyesight is affected due to extraction of teeth or not. Although the practice of informing by dentists is satisfactory, there is a need for creating awareness in the general public against such complications [14].

Majority of the individuals believed that dental extraction is the most painful procedure which included about 60% of the population. This was in accordance with the study conducted by Lakshmi *et al.* [13]. Some individuals believed that baby teeth should be thrown on the roof and some believed it should not be. The findings demonstrated the deprivation of the rural population, indicating the need for a focused program to disseminate scientific dentistry procedures to them. Policy makers and healthcare professionals should be commended in this regard for producing enough evidence [11]. About 60% of the respondents believed that extraction of tooth will lead to dental problems again and again to extract the other remaining teeth also which indicates about their belief that extraction is the cause leading them to other dental problems. Some believed that extracted tooth should be buried and some were against it, which points towards the age-old beliefs and misconceptions passed on from generations to generations. Majority of the individuals believed that extraction of root stumps is necessary and few believed that extraction should be done only if the root stump is painful. Fragments of retained roots are not unusual in the general population. Dentists and surgeons who routinely extract teeth often have a well-developed approach to removing retained roots to maintain clinical efficiency in the event of tooth fractures during surgery [15]. A surprising number of responders believed that head bath after dental extractions will increase the pain. Oral health education is very important and should be imparted to break these myths for the well-being of individuals in society. Most of the responders believed that extraction of upper teeth will lead to sinus problems and few were not sure for the same.51% believed that extraction is the only treatment of dental pain and 49% believed it is not the only treatment for dental pain. Most of the individuals believed that extraction should be done only when the tooth is shaking which indicates the lack of knowledge among the population.

59% believed that cheeks will appear hollow after extraction and similar percentage of people had a misconception about relieving dental pain with medicaments like Zandu Balm, Vicks or Iodex. So, it is the responsibility of dental care professions to educate the population about the proper treatment, to reduce the usage of homemade remedies and follow proper treatment modalities. Most of the responders thought that ageing is the only reason for extraction of teeth. Some believed that most of the time local anesthesia injections will not act in oral cavity; few believed that sometimes it may not act and few believed that it will act on oral cavity. Majority of responder had a point of view that sometimes nerves are affected due to injections and mostly lead to paralysis of face. Poor education, cultural beliefs and social misconceptions could be the reasons due to which myths are prevalent in the population and passed on to different generations. Based upon the present study, importance should be given for oral health education at individual as well as community level regarding the myths Co-ordinated efforts by dentists, Public Health Specialists, Non-Government Organisations (NGO's) and grass root level workers are needed to impart dental health education so that behavioural modification can increase the oral awareness and dental care utilization rate [16].

## Recommendations:

[1] There should be a regular education at individual as well as community level.

[2] There should be an agency that will ensure that public health education regarding myths and taboos among general population.

[3] Based upon the present study, importance should be given for oral health education at individual as well as community level regarding the myths Co-ordinated efforts by dentists, Public Health Specialists, Non-Government Organisations (NGO's) and grass root level workers are needed to impart dental health education so that behavioural modification can increase the oral awareness and dental care utilization rate.

## Conclusion:

The overall knowledge, attitude and their opinion regarding the taboos among Indians in rural area about dental extraction was average. Hence more comprehensive community teeth program should be implemented among general population.

## Consent:

A brief introduction about the study was given and informed consent was also taken from all the participants.

## Ethical approval:

As per international standard or university standard written ethical approval has been collected and preserved by the author(s).

## Competing interests:

Authors have declared that no competing interests exist.

## Figures and Tables

**Table 1:** Demographic details of study participants (N=100).

**Sr. No.**	**Demographic details**	**Responses**	**Number (N)**	**Percentage (%)**	**Total N (%)**
1	Age	18-30 Years	28	28	100
		31-45 Years	43	43	
		More than 45 Years	29	29	-100
2	Gender	Male	45	45	100 (100)
		Female	55	55	
3	Education	Educated	36	36	100 (100)
		Uneducated	64	64	
4	Occupation	Employed	26	26	100 (100)
		Unemployes	74	74	

**Table 2:** Opinion based question's responses of study participants (n=100).

**Sr. No.**	**Questions**	**Responses**	**Number (N)**	**Percentage (%)**	**Total**
1	1. Do you believe your eye sight will be affected due to extraction of teeth?	Yes	33	33	100 (100)
		No	29	29	
		I don't know	38	38	
2	Do you believe that dental extraction is the most painful procedure?	Yes	60	60	100 (100)
		No	40	40	
3	Do you believe that baby teeth should be thrown on the roof?	Yes	45	45	100(100)
4	4. Do you believe that extraction of tooth will lead you to dental problems again and again to extract the other remaining teeth also?	Yes	56	56	100(100)
		No	44	44	
5	Do you believe that extracted teeth should be burried?	Yes	53	53	100 (100)

**Table 3:** Practice/knowledge based question's responses of study participants

**Sr. No.**	**Questions**	**Responses**	**Number (N)**	**Percentage (%)**	**Total**
1	Do you think that extraction of root stump is not necessary?	Yes, Extraction not necessary	34	34	100(100)
		No, Extraction is necessary	46	46	
		Extraction necessary, only if it's painful	20	20	
2	Do you think head bath after dental extraction will increase the pain?	Yes	58	58	100 (100)
		No	42	42	
3	Do you think extraction of upper teeth will lead to sinus problem?	Yes	52	52	100 (100)
		No	22	22	
		I don't know	26	26	
4	Do you think extraction is the only treatment for dental pain?	Yes	51	51	100 (100)
		No	49	49	
5	Do you think extraction of the teeth should be done only when it is shaking?	Yes	66	66	100(100)
		No	34	34	
6	Do you think your cheeks will appear hollow after extraction?	Yes	59	59	100 (100)
		No	41	41	
7	Do you think application of any of the medicaments like Zandu Balm ,Vicks or Iodex will relieve dental pain?	Yes	59	59	100 (100)
		No	41	41	
8	Do you believe that ageing is only reason for extraction of teeth?	Yes	59	59	100 (100)
		No	41	41	
9	Do you think most of the time local anesthesia injections will not act in oral cavity?	Yes	44	44	100 (100)
		No	19	19	
		Some times	37	37	
10	Do you think your nerves will be affected due to injections and will mostly lead to paralysis of face?	Yes	35	35	100 (100)
		No	11	11	
		Sometimes	54	54	
